# The impact of tetrodotoxin (TTX) on the gut microbiome in juvenile tiger pufferfish, *Takifugu rubripes*

**DOI:** 10.1038/s41598-024-66112-y

**Published:** 2024-07-31

**Authors:** Mai A. Wassel, Yoko Makabe-Kobayashi, Md Mehedi Iqbal, Tomohiro Takatani, Yoshitaka Sakakura, Koji Hamasaki

**Affiliations:** 1 Atmosphere and Ocean Research Institute, The University of Tokyo, 5-1-5 Kashiwanoha, Kashiwa, Chiba 277-8564 Japan; 2 Genetics and Genetic Engineering Research Group, National Institute of Oceanography and Fisheries, NIOF, Cairo, Egypt; 3 Graduate School of Integrated Science and Technology, Nagasaki University, 1‑14 Bunkyo, Nagasaki, 852‑8521 Japan; 4 Collaborative Research Institute for Innovative Microbiology, The University of Tokyo, 1-1-1, Yayoi, Bunkyo-ku, Tokyo, 113-8657 Japan; 5 Department of Integrated Biosciences, Graduate School of Frontier Sciences, The University of Tokyo, 5-1-5 Kashiwanoha, Kashiwa, Chiba 277-8562 Japan

**Keywords:** Pufferfish *Takifugu rubripes*, Tetrodotoxin (TTX), Gut microbiome, Metabolic capabilities, Uncultured *Arcobacteraceae*, *Mycoplasma*, Molecular biology, Biodiversity, Molecular ecology, Sequencing

## Abstract

Tetrodotoxin (TTX) is a potent neurotoxin that accumulates in *Takifugu rubripes*, commonly known as pufferfish, through the ingestion of TTX-bearing organisms as part of their food chain. Although researchers believe that pufferfish use TTX to relieve stress, data are not currently available on how TTX affects the gut microbiota of pufferfish. To address this gap, our study aimed to investigate whether administering TTX to fish could alter their gut microbiota and overall health under various salinity conditions, including 30.0 ppt, 8.5 ppt, and 1.7 ppt salinity, which represent full-strength, isosmotic, and low-salinity stress, respectively. We analyzed the effect of TTX ingestion on the community structure, core microbiome, and metabolic capabilities of the gut microbiome using high-throughput sequencing technologies. The predominant bacterial taxa within the gut microbiome were Firmicutes (21–85%), Campilobacterota (2.8–67%), Spirochaetota (0.5–14%), and Proteobacteria (0.7–9.8%), with *Mycoplasma*, uncultured *Arcobacteraceae*, *Brevinema*, *Vibrio*, *Rubritalea,* and uncultured *Pirellulaceae* as core genera. Our findings indicated that the impact of TTX on high-abundance genera at 30.0 ppt and 8.5 ppt salinity levels was negligible, indicating their stability and resilience to TTX ingestion. However, at 1.7 ppt, TTX-fed fish showed a significant increase in uncultured *Arcobacteraceae.* Furthermore, our analysis of TTX-fed fish revealed taxonomic alterations in low-abundance taxa, which altered the predicted functions of the gut microbiota at all salinity levels. These results suggest that TTX administration could cause subtle effects on the metabolic functions of gut microbial communities. Overall, our study provides insights into the complex relationship between a TTX-accumulating animal, *T. rubripes*, and its gut microbiota*.*

## Introduction

Tetrodotoxin (TTX) is a potent neurotoxin found in marine organisms that poses a threat to human and animal health^1,2^. This neurotoxin blocks sodium ion channels in both the central and peripheral nervous systems, disrupting the normal flow of ions and resulting in paralysis, respiratory failure, and, in severe cases, death^3,4^. Due to its high toxicity, many countries and regions have imposed restrictions on consuming TTX-bearing organisms such as pufferfish^5,6^. TTX is produced by marine bacteria from genera such as *Vibrio*, *Aeromonas*, *Alteromonas*, *Bacillus*, *Pseudomonas*, *Actinomycetes*, and *Shewanella*^7,8^. These bacteria are found in various marine organisms, including pufferfish (*Chelonodon patoca*)^9^, toxic gobies (*Yongeichthys criniger*)^9^*,* xanthid crabs (*Atergatis floridus*)^10^, Pacific oysters (*Crassostrea gigas*)^11^, and toxic flatworm eggs (*Planocera multitentaculata*)^12^. Additionally, TTX may be present in environments such as sinking particles^13^ and sediments^14^.

*Takifugu rubripes*, commonly known as tiger pufferfish or Japanese fugu, is a marine species found in the northwest Pacific Ocean, particularly around Japan. It belongs to the order Tetraodontiformes and the family Tetraodontidae^15,16^. This fish is a model organism and holds great promise for aquaculture^17,18^. *T. rubripes* becomes toxic when it consumes TTX-bearing organisms, with this toxicity varying seasonally and in different parts of its body, including the liver, skin, intestines, and gonads^5,12,19–21^. However, it can become nontoxic in a controlled environment devoid of TTX-bearing organisms, which represents an interesting ecological adaptation^22,23^.

Like all vertebrates, *T. rubripes* harbors a diverse collection of microorganisms in its gut, known as the gut microbiome. This microbiome plays a crucial role in the physiological functions of fish, including metabolism, growth, development, and immune defense, effectively acting as an essential “extra organ”^24,25^. Therefore, maintaining healthy gastrointestinal tract homeostasis is critical for ensuring optimal fish health and growth^26^. Factors such as salinity and diet primarily influence this balance^27–30^.

Recent advances in high-throughput sequencing technologies have facilitated the exploration of the ecology, physiology and molecular functions of the microbiome^31,32^. Previous studies have provided valuable insights into the composition and dynamics of the gut microbiota in tiger pufferfish, laying the foundation for understanding their intricate microbial communities^27–29^. Notably, *Arcobacter* constituted a substantial portion (42.8%) of the total bacterial taxa at the genus level in the intestinal community^33^. The enrichment of *Enterococcaceae* in the intestinal bacterial community from recirculating aquaculture systems (RAS) and *Vibrionaceae* in offshore sea cage aquaculture systems (OSCS) highlights the influence of rearing environments on specific bacterial taxa^33^. Moreover, studies that have examined the dominant phyla in the intestine of tiger puffer, particularly Proteobacteria, Tenericutes and Firmicutes, offer a broader taxonomic perspective^27^. At the genus level, *Vibrio*, *Enterobacter*, *Bacillus*, *Pseudomonas*, *Exiguobacterium*, *Staphylococcus*, *Acinetobacter*, *Pseudoalteromonas*, and *Shewanella* have been identified in the intestines of young farmed pufferfish (*T. rubripes*), underscoring the diverse microbial landscape within their digestive tract^27,34^. Remarkably, *Vibrio*, *Enterobacter*, and *Bacillus* emerged as the most dominant genera, collectively accounting for 70.7% of the total composition^34^. Despite these comprehensive findings, no previous study has specifically investigated the impact of TTX ingestion on the gut microbiota community structure under varying salinity conditions. This gap in the literature emphasizes the novelty and significance of our study. By exploring the role of TTX, our research aimed to contribute valuable data to our understanding of the factors shaping the gut microbiota in juvenile pufferfish.

TTX plays a multifaceted role in the ecology and physiology of pufferfish (*T. rubripes*), influencing both their interactions with predators and their physiological responses to stress. Acting as a potent chemical defense mechanism, TTX protects pufferfish, particularly during their early life stages^35^. It has been found to act as a stress-relieving substance for pufferfish juveniles by reducing crowding stress and agonistic interactions among individuals^36^. Additionally, TTX is passed from the mother to her offspring in the eggs, providing protection to the larvae^37,38^. TTX has been shown to potentially possess antibacterial properties, capable of suppressing the growth of certain gram-positive and gram-negative bacteria^39,40^. However, some bacterial species, such as *Bacillus halodurans* and *Arcobacter butzleri*, are insensitive to TTX, even at micromolar toxin concentrations^41,42^. Despite its known effects on predators, stress relief, and antibacterial properties, the impacts of TTX on the gut microbiota community structure and metabolic functions have not been fully studied.

When juvenile *T. rubripes* migrate from offshore regions to estuaries, they encounter varying salinity levels that can stress them and their gut microbiota^43,44^. Our specific research questions are as follows: How does TTX administration affect the diversity and composition of the gut microbiota in tiger pufferfish under various salinity conditions? What metabolic functions are influenced by changes in the gut microbiota following TTX administration? We hypothesize that administering TTX to fish can alter their gut microbiota, leading to distinct changes in metabolic functions that can affect the host's physiology.

The objectives of this study are twofold: first, to characterize the gut microbiota community structure and core microbiome of cultured tiger pufferfish under different salinity conditions and, second, to identify shifts or alterations in the community structure induced by TTX administration by comparing the control fish with the TTX-fed fish.

## Materials and methods

### Experimental fish

In June 2021 and June 2022, two batches of nontoxic juveniles of *T. rubripes* were acquired from Nagasaki Fishery Public Corporation in Sasebo, Nagasaki, Japan. These hatchery-reared juveniles were then transferred to the Graduate School of Integrated Science and Technology, Nagasaki University. A total of 120 fish were stocked in a 120-L cylindrical tank with a recirculating aquaculture system (RAS) at a salinity of 34.0 ppt. The salinity of 34.0 ppt was maintained by dissolving artificial seawater (Marine Art Hi, Tomita Pharmaceutical, Tokushima, Japan) in dechlorinated tap water. To ensure optimal conditions, the tank was placed in a temperature-controlled room set at 25 °C, with pure oxygen supplied. The fish were acclimatized by feeding a control diet (TTX-free diets; Otohime S1 (2021; 1.0 mm in diameter) and Otohime C1 (2022; 580 to 840 µm in diameter); Marubeni Nisshin Feed, Tokyo, Japan) twice daily until satiation for several days.

### Preparation of the TTX-containing diet

The TTX-containing diet (2.45 mouse units (539 ng)/g diet) and the nontoxic test diet were prepared according to Amano et al.^36^. The TTX-containing emulsion and control emulsion were sprayed onto 250 g of diet material to prepare the TTX-containing diet and the non-toxic test diet, respectively. The concentration of adsorbed TTX was measured in a subsample of the diets, as described in Sakakura et al.^45^. Based on the measurement of adsorbed TTX, the effective concentration of TTX in the diet was determined to be 539 ng/g of diet.

### Rearing experiment

The experimental procedure for controlling the salinity and diet of *T.* *rubripes* juveniles is described in Table 1 and Supplementary Fig. 1. The Fish were initially obtained from the stock tank with a salinity of 34.0 ppt and then anesthetized with 100 ppm MS222 (Finquel MS 222, Sigma, Inc.). Their standard length (SL) in millimeters and body weight (BW) in grams were measured using a digital caliper (CD20-GM; Mitsutoyo Cooperation, Kanagawa, Japan) and an electric balance (PB153-S; Mettler-Toledo, OH, USA), respectively, with a precision of up to one decimal point. The juveniles had average standard lengths of 53.7 mm (2021) and 49.8 mm (2022) and average body weights of 4.4 g (2021) and 4.0 g (2022). In the 2021 rearing experiment, on July 4, 2021 (designated Day 0), a total of 80 fish were transferred to four 200-L black polyethylene tanks, with 20 fish in each tank. These tanks had a recirculating system (approximately 50 L/h), and the fish were acclimatized to 8.5 ppt salinity to establish isosmotic conditions. Beginning on July 16 (Day 12), the salinity in the tanks was gradually decreased from 8.5 to 5.1 ppt (one tank for each feeding group). Beginning on July 18 (Day 14), the salinity in the tanks was gradually decreased from 5.1 to 1.7 ppt (one tank for each feeding group). The fish were reared at the corresponding salinities until the end of the experimental period (Day 20). The TTX-containing diet was administered to the TTX-diet group (in two tanks), while the nontoxic test diet was fed to the control group (in two tanks), both at 9:00 and 15:00. In the 2022 rearing experiment, a total of 40 fish were transferred to two 200-L black polyethylene tanks, with 20 fish in each tank. These fish were reared at 30.0 ppt salinity for eight days, with or without the TTX-containing diet.

**Table 1:**
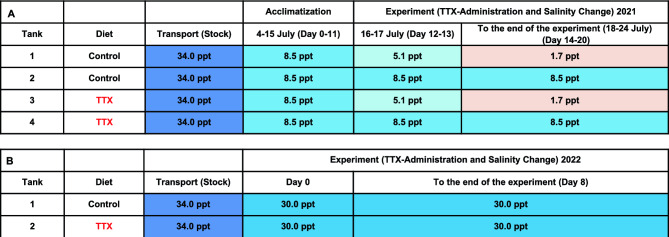
Experimental procedure for controlling the salinity and diet of *T.* *rubripes* juveniles.

(**A**) Experiment (TTX-Administration and Salinity Change) 2021, 8.5 ppt (isosmotic) and 1.7 ppt (low- salinity stress); (**B**) Experiment (TTX-Administration and Salinity Change) 2022, 30.0 ppt (full-strength seawater).

### Sample collection

At the end of the 2021 rearing experiment, 6 to 10 juvenile *T. rubripes* individuals were randomly selected and evaluated. Their SL and BW were measured after administering anesthesia with 200 ppm MS222 (Finquel MS 222, Sigma, Inc.) (Supplementary Table 1). Blood was collected from the sinus venosus using a heparinized syringe to measure plasma osmolality, cortisol levels, and lysozyme levels. Blood samples were then centrifuged at 2500×*g* for 15 min at 4 °C (Model 7780, KUBOTA, Tokyo, Japan). The plasma analysis was conducted by following the method outlined in Sakakura et al.^45^. The concentrations of TTX in the liver, muscle, skin, and tissue of both the control and TTX-fed fish groups were determined using an LC‒MS/MS method, as previously described^46,47^.

At the end of the 2022 rearing experiment, 13–16 juvenile *T. rubripes* specimens were randomly collected and assessed. Similar to previous procedures, their SL and BW were measured after administering anesthesia with 200 ppm MS222 (Supplementary Table 1). Their intestinal tracts were aseptically removed and stored at − 80 °C until subsequent processing. The gut contents were evacuated using a syringe filled with Tris–EDTA buffer (100 mM Tris–HCl, 200 mM EDTA, pH 8) into sterile propylene tubes. The samples were then preserved at − 20 °C until further processing for molecular analysis^48,49^.

### DNA extraction and PCR amplification of the bacterial 16S rRNA gene

DNA was extracted from the gut contents of each individual using the ISOIL DNA Isolation Kit (Nippon Gene Co., Ltd., 316-06211, Japan) according to the manufacturer's protocol with appropriate modifications. Chemical lysis was initiated by combining the lysis solution HE, enzymatic lysis buffer (20 mM Tris–HCl, pH 8.0, 2 mM EDTA, and 1.2% Triton X-100), and 200 mg/mL lysozyme, followed by incubation at 37 °C for 30 min. After lysozyme treatment, 50 μL of proteinase K (20 mg/mL) and 60 μL of lysis buffer 20S were added and thoroughly mixed. The samples were then incubated at 55 °C for 30 min with gentle shaking. After the incubation, the samples were centrifuged at 8000 rpm for 15 min, and the supernatant was transferred to a new 5 mL tube. A mixture of chloroform and purification solution was added to the supernatant and carefully mixed. After centrifugation at 9000 rpm for 15 min, the upper aqueous phase was carefully transferred to a new 5 mL tube. Precipitation solution was added, and the solution was thoroughly mixed. Further centrifugation was performed at 9000 rpm for 1 h, after which the supernatant was removed. The remaining pellet was washed twice with 500 μL of wash buffer. Then, ice-cold ethanol (70%) and Ethachinmate were added to the sample and gently inverted several times. The mixture was incubated at room temperature for 5 min. After centrifugation at 10,000 rpm for 15 min, the supernatant was removed, and 250 μL of ice-cold 95% ethanol was added to the pellet. Following another round of centrifugation at 10,000 rpm for 5 min, the ethanol was carefully removed, and the pellet was air-dried at room temperature for approximately 15–20 min until the ethanol evaporated. Subsequently, the pellet was resuspended in 50 μL of TE buffer (10 mM Tris–Cl, 0.5 mM EDTA; pH 8.0) provided by ISOIL. A total of 2 μL of RNase (100 mg/mL) (Thermo Fisher Scientific, Rochester, USA, EN0551) was added to enzymatically remove any remaining RNA, and the mixture was incubated at 37 °C for 10 min. The concentration of the purified metagenomic DNA was measured using a Qubit 2.0 fluorometer (Thermo Fisher Scientific, Rochester, USA) and a Qubit™ dsDNA HS Assay Kit (Invitrogen, Q32851). DNA quality was assessed using a Nanodrop spectrophotometer according to the manufacturer’s protocol and 1% agarose gel electrophoresis. The gel was stained with 5 μL/100 mL of RedSafe™ nucleic acid staining solution (20,000×) (iNtRON Biotechnology, 21,141) and imaged using a gel documentation system (BioDoc-it).

In 2021, 36 samples were prepared for PCR amplification and next-generation sequencing (NGS) using the Illumina MiSeq platform. In 2022, 29 samples were prepared for the same purpose. The 16S rRNA gene was amplified in a PCR mixture using the primers 515F (5′-GTGCCAGCMGCCGCGGTAA-3′) and 806R (5′-GGACTACHVGGGTWTCTAAT-3′), which target the V4 hypervariable region^50^. These V4 regions were chosen due to their high resolution and lower bias in detecting bacterial taxa^51,52^. A KAPA HiFi HotStart ReadyMix PCR Kit (KAPA Biosystems, Boston, MA, USA) was used for PCR amplification according to the manufacturer’s protocol. PCRs were conducted in triplicate using a 25 μL volume, which included 5 μL (60 ng/μL) of diluted template DNA, 0.7 μL (5 μM) each of the forward and reverse primers, 6.1 μL of PCR-grade water, and 12.5 μL of 2 × KAPA HiFi HotStart ReadyMix. Thermal cycling was performed for 28 cycles under the following conditions: initial denaturation at 94 °C for 3 min, denaturation at 94 °C for 30 s, annealing at 60 °C for 45 s, extension at 72 °C for 1 min, and a final extension at 72 °C for 5 min. After PCR amplification, the presence of PCR products and their sizes were confirmed via 1.5% agarose gel electrophoresis. Three technical PCR replicates from each sample were pooled in a 0.5 mL PCR tube to minimize PCR bias. The PCR products were purified using the QIAquick^®^ Gel Extraction Kit (Qiagen, Cat. No. 28706, USA). The purified DNA was then subjected to a quality analysis through electrophoresis on a 1.5% agarose gel and quantified using the Qubit™ dsDNA HS Assay Kit (Invitrogen, Q32851).

### Library construction and Illumina amplicon sequencing

Library preparation was performed using the Nextera^®^ XT Index Kit v2 (Illumina, San Diego, CA, USA) Set A (96 indices, 384 samples) according to the manufacturer’s protocol. Index PCR was conducted to attach dual indices and Illumina sequencing adapters. The reaction mixture included 12.5 μL of 2 × KAPA HiFi HotStart ReadyMix (0.5 U), 2.5 μL of Nextera^®^ XT Index Primer 1 (N7xx), and 2.5 μL of Nextera^®^ XT Index Primer 2 (S5xx) (200 nM) with the purified PCR product in each sample. The reaction volume of 25 μL per sample was adjusted with PCR-grade water. The PCR amplification process began with an initial denaturation step at 95 °C for 3 min, followed by 8 cycles of denaturation at 95 °C for 30 s, annealing at 55 °C for 30 s, and extension at 72 °C for 30 s, prior to a final extension step at 72 °C for 5 min. Subsequently, the PCR products were cleaned using AMPure XP beads (Beckman Coulter Inc., Beverly, Massachusetts, USA). The 16S rRNA amplicon libraries for each sample were quantified using the Qubit™ dsDNA HS Assay Kit (Invitrogen, Q32851). PCR samples were pooled at equimolar concentrations of 2 nM. The quality of the pooled samples was assessed using an Agilent 2100 Bioanalyzer system with High Sensitivity DNA Chips (Agilent Technologies, Germany). The pooled libraries were denatured with 0.2 N NaOH and diluted with prechilled HT1 buffer (hybridization buffer) to a final concentration of 4 pM. Sequencing was performed using a MiSeq Reagent Kit v3–600 cycle (Illumina, San Diego, CA, USA) according to the manufacturer’s protocols. Paired‐end sequencing was performed on an Illumina MiSeq platform with a 5% PhiX spike‐in quality control using the PhiX Control Kit v3 (Illumina, San Diego, CA, USA).

### Data analysis

The software package Quantitative Insights into Microbial Ecology 2 (QIIME2, Version 2022.2, https://qiime2.org/) was used to analyze the raw amplicon sequence data by following the methods outlined in Iqbal et al.^53^. Initially, the raw paired-end FASTQ reads were demultiplexed using the Cutadapt plug-in^54^ and imported into QIIME2 in Casava 1.8 demultiplexed (paired-end) format. Subsequently, the demultiplexed reads underwent quality filtering, denoising, chimera checking, and dereplication using the DADA2 (Divisive Amplicon Denoising Algorithm 2)^55^ denoise-paired plugin. Before performing the analysis, the quality plots of forward and reverse reads were evaluated. Taxonomic affiliation was determined using the QIIME feature classifier classify-sklearn on the SILVA-138^56^. The MAFFT algorithm^57^ was used for alignment, and a phylogenetic tree was constructed using FastTree software^58^. This tree was then used for the UniFrac distance analysis^59^. Furthermore, the amplicon sequence variant (ASV) table obtained from DADA2 was filtered to eliminate sequences with low repetition and sequences classified as organisms other than bacteria (e.g., chloroplasts, mitochondria, and unassigned). The sequences were rarefied to 23,287 reads using the feature-table rarefy function^60^. Rarefaction curves (Supplementary Fig. 9) were generated to assess the sequencing depth and compare relative microbial richness among different samples based on the observed number of ASVs and the Shannon alpha diversity index.

### Data visualization and statistical analyses

The growth data for each group were analyzed for equal variance and normality using the Bartlett test and Shapiro‒Wilk test, respectively. For data meeting the parametric criteria, a two-way analysis of variance (2-way ANOVA) was conducted, followed by Tukey’s honestly significant difference (HSD) test^61^ to examine the effects of oral TTX administration (factor ‘diet’) and chronic changes in salinity (‘salinity’). Conversely, for nonparametric data, an aligned rank transform (ART) for nonparametric factorial ANOVA^62^ was employed, followed by Dunn’s test with the Bonferroni correction for the statistical analysis of the effects of diet and salinity. The plasma osmolarity, plasma cortisol, and plasma lysozyme data were analyzed in the same manner. All the statistical analyses were performed in the R environment^63^ with the ‘ARTool’^62^, and ‘vegan’^64^ packages, and *p* values < 0.05 were considered to indicate statistical significance in all the analyses.

Community structure and composition analyses were conducted by processing the ASV table in the R environment using several packages, including ‘phyloseq’^65^, ‘microbiome’^66^, ‘vegan’, and ‘ggplot2’^67^. Alpha diversity indices, including the Chao1^68^ and Shannon indices^69^, were calculated in R using the ‘vegan’ package. Two-way analysis of variance (2-way ANOVA) was performed in R to evaluate the significance of alpha diversity matrices (Chao1 and Shannon) among the salinity levels (30.0, 8.5, and 1.7). Subsequently, Tukey’s honestly significant difference (HSD) test was applied to examine pairwise group differences. Beta diversity was computed in R to compare the microbial community composition among all samples at different salinity levels. Initially, UniFrac dissimilarities (weighted) were computed using the ‘phyloseq’ package and visualized through principal coordinate analysis (PCoA) and nonmetric multidimensional scaling (NMDS). Then, two-way PERMANOVA was performed using the ‘Adonis’ function from the ‘vegan’ package with 999 permutations. The relative abundance of each taxon at the genus level was compared between control fish and TTX-fed fish using analysis of variance (ANOVA). Shared and unique gut ASVs were visualized using Venn diagrams through the web-based tool Venny 2.1.0^70^. A heatmap was constructed using the ‘devtools’^71^, ‘qiime2R’^72^, and ‘tidyverse’^73^ packages in R. Additionally, a heatmap depicting the core microbiome of the control fish was generated using MicrobiomeAnalyst 2.0^74^. Linear discriminant analysis effect size (LEfSe) was used to identify taxonomic differences between control fish and TTX-fed fish at different salinity levels, with a *p* value of 0.05 and an LDA score of 3.5^75^.

### Prediction of microbial functions

Tax4Fun2^76^, a tool available in MicrobiomeAnalyst 2.0, was used to predict the functional profile of gut bacterial communities based on 16S rRNA gene sequencing data. The relative abundance of functions of the DNA was normalized to the copy number of orthologous groups according to the Kyoto Encyclopedia of Genes and Genomes (KEGG) pathway database^77^. KEGG metabolism was used to generate an interactive heatmap using R. The functional profile analysis was performed using STAMP software (version 2.1.3)^78^ at 95% confidence intervals to evaluate the significance of functions among the salinity levels.

### Ethics statement

All animal procedures were performed in compliance with the ARRIVE guidelines and were approved by the Animal Care Committee of Nagasaki University. The study protocol underwent review and approval by the committee to ensure the humane treatment and welfare of the animals. All procedures and animal handling were conducted in accordance with the approved guidelines, with a primary focus on minimizing potential distress or harm. Special attention was given to maintaining appropriate housing conditions, providing proper nutrition, and ensuring overall care throughout the experiment.

## Results

### Measurement of physiological parameters

A significant difference in body weight was observed between groups treated with different levels of salinity (ART for nonparametric factorial ANOVA, *df* = 2, *F* = 175.3, *p* = 2e−16). Between groups fed different diets, no significant difference in body weight was found (*df* = 1, *F* = 1.213, *p* = 0.275). A significant interaction between the two factors ‘diet’ and ‘salinity’ was not observed (*df* = 2, *F* = 0.951, *p* = 0.392). At a salinity level of 1.7 ppt, the body weight of the TTX-fed fish was significantly greater than at 8.5 ppt salinity (Dunn's test, *p* = 0.032) (Supplementary Fig. 2A). The median plasma osmolality at both 1.7 ppt and 8.5 ppt was approximately 342 mOsm/kg, which is within the physiological range for *T. rubripes* (Supplementary Fig. 2B). Additionally, in fish reared at 1.7 ppt salinity, plasma cortisol levels were significantly higher than in those reared at 8.5 ppt salinity (two-way ANOVA, 'salinity', *df* = 1, *F* = 6.208, *p* < 0.05). No significant differences in plasma cortisol levels were found between the ‘diet’ factor (*df* = 1, *F* = 4.130, *p* = 0.051) or the interaction between ‘diet’ and ‘salinity’ (*df* = 1, *F* = 0.193, *p* = 0.793) (Supplementary Fig. 2C). The study also revealed a significant difference in plasma lysozyme activity between ‘diet’ (2-way ANOVA, *df* = 1, *F* = 5.910, *p* = 0.020), but no significant difference was found between ‘salinity’ (2-way ANOVA, *df* = 1, *F* = 1.594, *p* = 0.214) or the interaction between ‘diet’ and ‘salinity’ (2-way ANOVA, *df* = 1, *F* = 0.191, *p* = 0.664) (Supplementary Fig. 2D). In most of the tested fish, TTX was not detected; however, trace amounts were detected in the skin (0.2 MU/g) and tissue (0.6 MU/tissue) of TTX-fed fish reared at 1.7 ppt salinity (n = 9, Supplementary Table 2).

### Community structure of the gut microbiota of juvenile *Takifugu* rubripes

**Figure 1:**
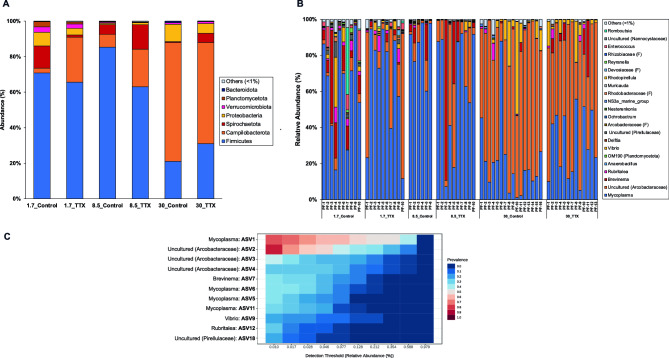
The gut bacterial communities of *T. rubripes* juveniles were analyzed in two groups—Control fish and TTX-fed fish—at three different salinity levels: 1.7 ppt (low-salinity stress), 8.5 ppt (isosmotic), and 30.0 ppt (full-strength seawater). (**A**) Mean relative abundance of the gut bacterial community at the phylum level; (**B**) Relative abundance of the gut bacterial community at the genus level. Groups constituting less than 1% of the total community are combined and referred to as “Others (< 1%)”. Four taxa, Rhizobiaceae, Devosiaceae, Rhodobacteraceae, and Arcobacteraceae, are shown at the family level; and (**C**) Core bacterial taxa of the gut bacterial community in control fish at the ASV level, based on an abundance threshold (> 0.01%) for taxa with a prevalence greater than 0.9. The x-axis represents the detection thresholds, with lower abundance values on the left and higher abundance values on the right. The color shading represents the prevalence of each bacterial genus among the samples for each abundance threshold. As the detection threshold increases, the prevalence decreases.

High-throughput sequencing of intestinal tract samples from juvenile *T. rubripes* resulted in 5,924,027 reads, with a mean read depth of 75,423 sequences per sample. A total of 1,235 ASVs were detected during the initial identification, with an appearance frequency of 4,902,468 across all samples. The gut microbiota of *T. rubripes* juveniles exhibited 18 phyla, with seven phyla having an abundance greater than 1%. Firmicutes was the most dominant phylum, ranging from 21 to 85% across the different salinity levels. The second most dominant phylum was Campilobacterota, with an abundance varying from 2.8 to 67%. Spirochaetota (0.5–14%) and Proteobacteria (0.7–9.8%) were also well represented, while the remaining phyla, including Verrucomicrobiota, Planctomycetota, and Bacteroidota, accounted for no more than 14% (Fig. 1A). At the genus level, the top five taxa were *Mycoplasma* (21–85%), uncultured *Arcobacteraceae* (2.7–67%), *Brevinema* (0.5–14%), *Vibrio* (0.2–8.2%), and *Rubritalea* (0.2–2.8%), which collectively constituted approximately 96% of the total abundance (Fig. 1B). Some variation was observed among individuals within each group. *Mycoplasma,* uncultured *Arcobacteraceae, Brevinema, Vibrio, Rubritalea,* and uncultured *Pirellulaceae* were identified as core bacterial taxa. The detection thresholds of 0.01% and 0.321% revealed a high prevalence of *Mycoplasma* and uncultured *Arcobacteraceae* in the gut microbiome of *T. rubripes* juveniles (Fig. 1C).

### Differences in the gut microbiota between juvenile pufferfish fed control diets and those fed TTX-containing diets

Following TTX administration, the gut microbiome remained predominantly dominated by Firmicutes and Campylobacterota across diverse salinity conditions. At lower salinity levels (8.5_TTX and 1.7_TTX), TTX administration resulted in an increased abundance of Campilobacterota compared to that in the control diet groups, with abundances changing from 7.1 to 21% and from 2.8 to 25%, respectively (Fig. 1A). At the genus level, *Mycoplasma* was prevalent in the gut microbiome under low-salinity conditions, with a high abundance in the 1.7_TTX group (65%), followed by the 8.5_TTX group (63%), while it exhibited a low abundance in the gut microbiome of the 30_TTX group (31%). Conversely, uncultured *Arcobacteraceae* remained the dominant genus in the gut microbiome of fish reared at relatively high salinity levels (30_TTX), with a high abundance of 57% (Fig. 1B).

**Figure 2:**
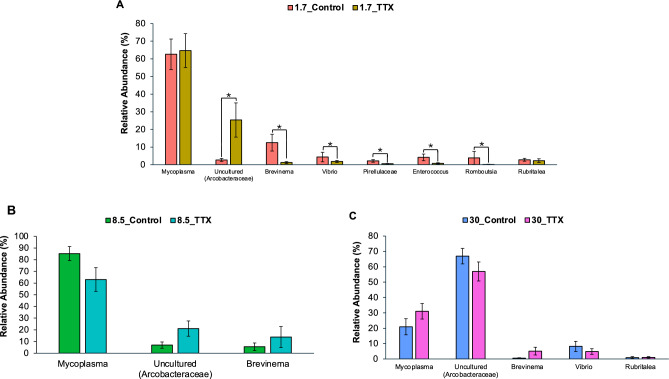
Relative abundances of major intestinal bacterial taxa derived from control fish and TTX-fed fish are presented at the genus level. Panels depict comparisons between (**A**) 1.7_Control & 1.7_TTX groups; (**B**) 8.5_Control & 8.5_TTX groups; and (**C**) 30_Control & 30_TTX groups. Each column is color-coded based on the treatment. Genera with a mean abundance > 1% are displayed. The bars represent the standard errors of the means. Asterisks (*) indicate significant differences (ANOVA, *p* < 0.05).

In TTX-fed fish at a salinity level of 1.7 ppt, statistical analyses revealed microbial instability, with a significant increase in the abundance of uncultured *Arcobacteraceae* and significant decreases in the abundances of *Brevinema*, *Vibrio*, uncultured *Pirellulaceae*, *Enterococcus*, and *Romboutsia* (ANOVA, *p* < 0.05) (Fig. 2A). At 8.5 ppt and 30.0 ppt salinity, no significant differences in the high-abundance genera were observed (Fig. 2B,C). Notably, the abundance of the genus *Mycoplasma* did not differ significantly between control fish and TTX-fed fish at any salinity level (Fig. 2).

**Figure 3:**
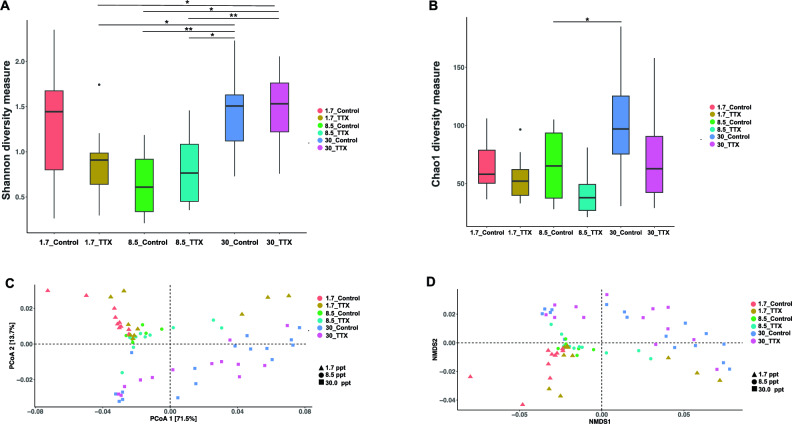
Alpha and beta diversity of control fish and TTX-fed fish. (**A**) Shannon diversity measure; (**B**) Chao1 diversity measure. Boxplots illustrate the distribution of these diversity indices. Asterisks denote significant differences (Tukey’s HSD test, *p* < 0.05 (*), *p* < 0.01 (**)); (**C**) Principal Coordinate Analysis (PCoA); and (**D**) Nonmetric Multidimensional Scaling (NMDS) were performed to visualize the structural differences in the microbial communities based on weighted UniFrac distance matrices. Samples are color-coded by sample type (salinity, diet), and different shapes are used to represent salinity levels (30.0 ppt, 8.5 ppt, and 1.7 ppt).

Alpha diversity analysis revealed no significant differences between control fish and TTX-fed fish at the same salinity level (Tukey’s HSD test, *p* > 0.05). After TTX administration, Chao1 indices indicated a decreasing trend in the gut microbiota, but this difference was not statistically significant (Tukey’s HSD test, *p* > 0.05) (Fig. 3A,B). The beta diversity analysis revealed no distinct differences in the gut bacterial communities between control fish and TTX-fed fish at the same salinity level. At a salinity level of 1.7 ppt, a slight, though not statistically significant, difference was observed between control fish and TTX-fed fish (PERMANOVA, *p* = 0.056) (Fig. 3C,D).

The heatmap depicts the 50 genera with the greatest differences between control fish and TTX-fed fish individuals at their respective salinity levels. In the control fish at 1.7 ppt, the relative abundances of *Rhizobium, Brevinema, Arcobacteraceae, Mesorhizobium, Romboutsia, Reyranella, Legionella, Gimesiaceae, Enterococcus,* and uncultured *Pirellulacea* were higher, while the relative abundances of *Rubinisphaera*, *Devosiaceae*, *Aeromonas*, and uncultured *Arcobacteraceae* were higher in the TTX-fed fish. At 8.5 ppt, *Devosiaceae, Arcobacteraceae*, and *Shinella* were more abundant in the control fish, while *Brevinema, Microbacteriaceae, Gimesiaceae*, and uncultured *Arcobacteraceae* exhibited higher relative abundances in the TTX-fed fish. Additionally, at 30.0 ppt, *Rhodobacteraceae*, *Ochrobactrum*, *Delftia*, *Tenacibaculum, Marivita*, *Legionellaceae*, *Nautella*, and *Nesterenkonia* were more abundant in the control fish, while *Brevinema* exhibited a higher relative abundance in the TTX-fed fish (Supplementary Fig. 3).

**Figure 4:**
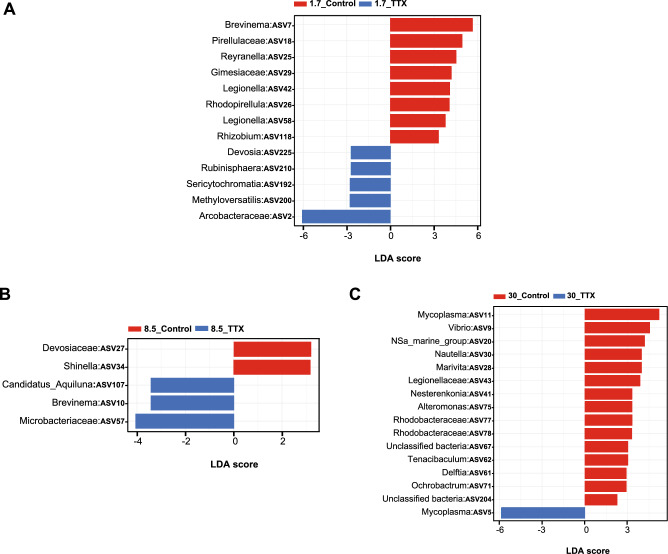
Linear discriminant analysis effect size (LEfSe) of microbial abundance in two groups—Control fish and TTX-fed fish—at three different salinity levels: (**A**) 1.7_Control & 1.7_TTX groups; (**B**) 8.5_Control & 8.5_TTX groups; and (**C**) 30_Control & 30_TTX groups. Only ASV-level taxa with significant differences at each salinity level were detected by LEfSe with an LDA threshold score of 3.5 and a *p* value of 0.05. Seven ASVs, Pirellulaceae, Gimesiaceae, Arcobacteraceae, Devosiaceae, Microbacteriaceae, Legionellaceae, and Rhodobacteraceae, are shown at the family level.

LEfSe identified significant enrichments of ASVs. At a salinity level of 1.7 ppt, the top 8 enriched ASVs in control fish were *Brevinema, Pirellulaceae, Reyranella, Gimesiaceae,* two ASVs of *Legionella, Rhodopirellula,* and *Rhizobium.* In TTX-fed fish at the same salinity level, the top 5 enriched ASVs were *Devosia*, *Rubinisphaera*, *Sericytochromatia*, *Methyloversatilis*, and *Arcobacteraceae* (Fig. 4A). Similarly, in the control fish reared at a salinity of 8.5 ppt, the top 2 enriched ASVs were *Devosiaceae* and *Shinella*, while the top 3 enriched ASVs in the 8.5_TTX-fed fish were *Candidatus Aquiluna*, *Brevinema*, and *Microbacteriaceae* (Fig. 4B). Additionally, 15 significantly enriched ASVs were observed in the 30_Control group. The top 5 enriched ASVs were *Mycoplasma, Vibrio, NSa marine group, Nautella,* and *Marivita.* Conversely, in 30_TTX-fed fish, the most enriched ASV was *Mycoplasma* (Fig. 4C).

**Figure 5:**
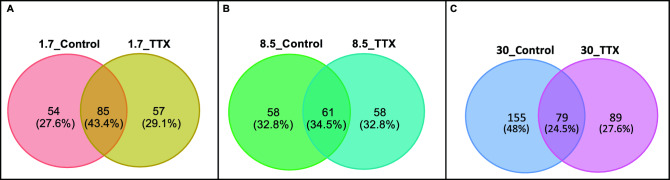
Venn diagram illustrating the shared and unique bacteria at the ASV level among control fish and TTX-fed fish reared at three different salinity levels: (**A**) 1.7_Control & 1.7_TTX groups; (**B**) 8.5_Control & 8.5_TTX groups; and (**C**) 30_Control & 30_TTX groups.

A Venn diagram was generated to visualize the shared and unique ASVs in each sample. At 1.7 ppt, 85 shared ASVs were identified between the control and TTX-fed fish groups, constituting 43.4%. In the 1.7_Control group, 54 unique ASVs were identified, and 57 ASVs were found in the 1.7_TTX group (Fig. 5A). Among the shared genera, *Mycoplasma,* uncultured *Arcobacteraceae, Brevinema,* and *Vibrio* were the most prevalent. *Reyrenella,* was a unique genus detected in the control fish, while *Cetobacterium* and *Enterococcus* were the predominant and unique ASVs in the 1.7_TTX treatment group (Supplementary Fig. 4A). Similarly, at 8.5 ppt, 61 shared ASVs were identified between the control and TTX-fed fish groups, accounting for 34.5% (Fig. 5B). Among the shared genera, *Mycoplasma,* uncultured *Arcobacteraceae*, and *Brevinema* were the most prevalent. *Shinella* and *Devosiaceae* were the top ASVs in the 8.5_Control group, while *Microbacteriaceae, Brevinema,* and *Candidatus Aquiluna* were the predominant ASVs in the 8.5_TTX group (Supplementary Fig. 4B). At a salinity level of 30.0 ppt, a total of 155 (48%) ASVs were identified in the control fish group, whereas 89 ASVs (27.6%) were detected in the TTX-fed fish group. Seventy-nine ASVs (24.5%) were shared between these two groups (Fig. 5C). Among the shared genera, *Mycoplasma,* uncultured *Arcobacteraceae*, *Vibrio,* and *Brevinema* were prevalent. In the 30_Control group, *Marivita* and *Vibrio* were the top ASVs, while *Rhodobacteraceae* and *Pseudoalteromonas* were the predominant ASVs in the 30_TTX group (Supplementary Fig. 4C).

### Differences in the predicted functions of the gut microbiota between juvenile pufferfish fed control diets and those fed TTX-containing diets

**Figure 6:**
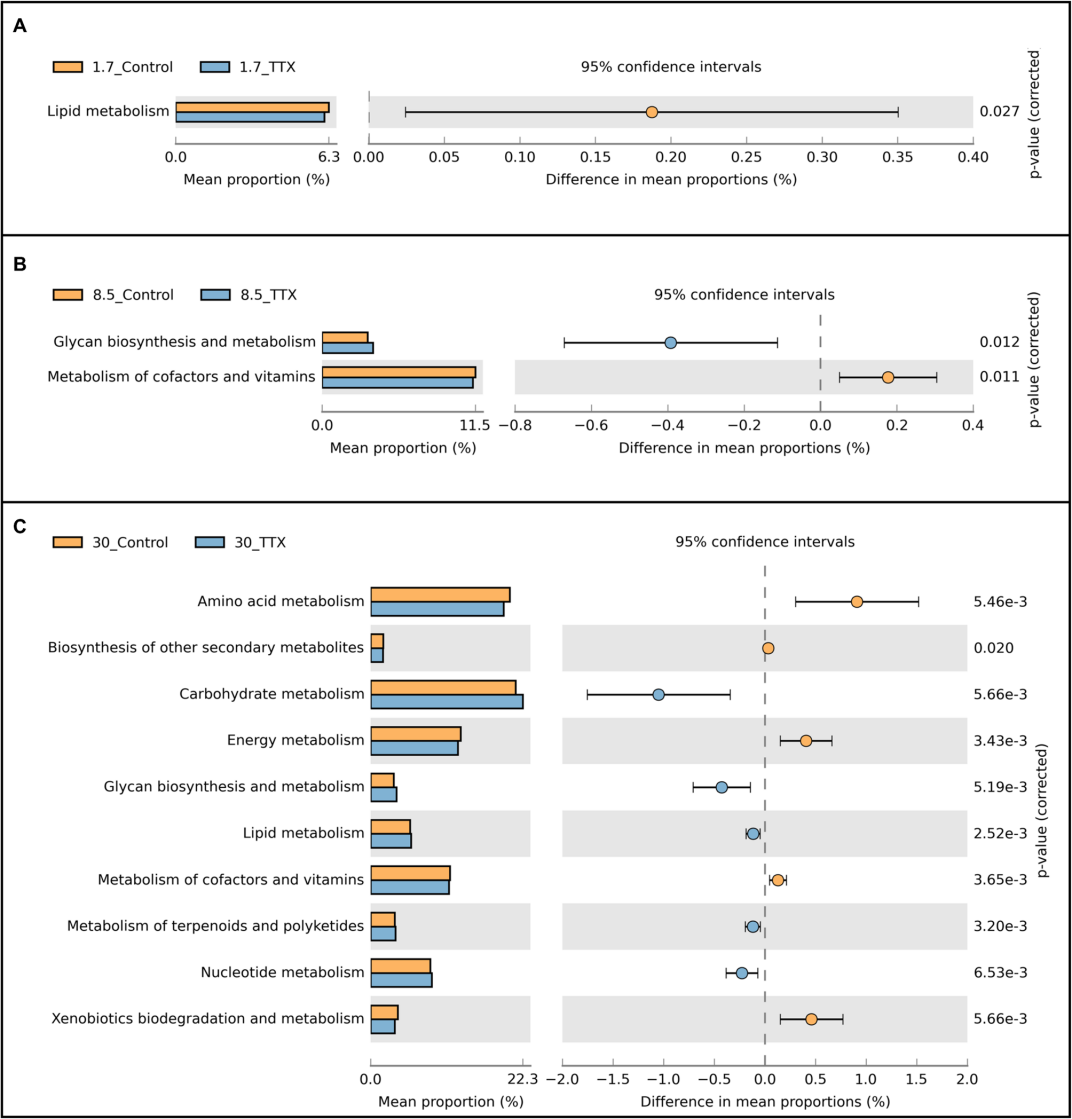
Relative abundance of the KEGG categories (level 2) of the bacterial community in control fish and TTX-fed fish at three different salinity levels: (**A**) 1.7_Control & 1.7_TTX groups; (**B**) 8.5_Control & 8.5_TTX groups; and **(C)** 30_Control & 30_TTX groups (Welch’s *t* test, *p* < 0.05). https://www.kegg.jp/kegg/kegg1.html.

The functional analysis using Tax4fun focused on the "Metabolism" category at level 3. The analysis revealed 11 categories at level 2 at three different salinity levels in fish fed diets with and without TTX, as shown in Supplementary Fig. 5. The gut microbiome showed significant changes at the 1.7 ppt salinity level, leading to alterations in predicted functions (Supplementary Fig. 5A). The “Lipid metabolism” category showed significant differences between the control and TTX groups (Welch’s *t* test, *p* < 0.05), with a higher abundance in the gut microbiota of control fish (Fig. 6A). Thirty-six pathways showed significant differences between the 1.7_Control and 1.7_TTX groups. Thirteen of 36 pathways were more abundant in the control group (Welch’s *t* test, *p* < 0.05). Conversely, 23 pathways were more abundant in the gut microbiota of the TTX group (Welch’s *t* test, *p* < 0.05) (Supplementary Fig. 6). Similarly, changes were observed at 8.5 ppt salinity, leading to significant alterations in the predicted functions (Supplementary Fig. 5B). The “Metabolism of cofactors and vitamins" and "Glycan biosynthesis and metabolism” categories showed significant differences between the control and TTX groups (Welch’s *t* test, *p* < 0.05). The control group showed a higher abundance of “Metabolism of cofactors and vitamins”, while the TTX group showed a higher abundance of “Glycan biosynthesis and metabolism” (Fig. 6B). A total of 15 pathways exhibited significant differences between the 8.5_Control and 8.5_TTX groups. Nine of 15 pathways were more abundant in the gut microbiota of the control group (Welch’s *t* test, *p* < 0.05). Conversely, 6 pathways were more abundant in the gut microbiota of the TTX group (Welch’s *t* test, *p* < 0.05) (Supplementary Fig. 7). Finally, shifts in the microbial community composition at 30.0 ppt salinity led to significant changes (Supplementary Fig. 5C). Ten categories, including “Amino acid metabolism,” “Biosynthesis of other secondary metabolites,” “Energy metabolism,” “Metabolism of cofactors and vitamins,” and “Xenobiotic biodegradation and metabolism”, showed significant differences (Welch’s *t* test, *p* < 0.05), with the control group showing higher abundance. “Carbohydrate metabolism,” “Glycan biosynthesis and metabolism,” “Lipid metabolism,” “Metabolism of terpenoids and polyketides,” and “Nucleotide metabolism,” also showed significant differences, with the TTX group showing higher abundance (Welch’s *t* test, *p* < 0.05) (Fig. 6C). A total of 105 pathways exhibited significant differences between the 30_Control and 30_TTX groups, with the top 74 KEGG categories (level 3) displaying high relative abundance. The gut microbiota of the control group had higher abundances of 11 pathways related to “Amino acid metabolism”, 5 pathways related to “Biosynthesis of other secondary metabolites”, 6 pathways related to “Energy metabolism”, 10 pathways related to “Metabolism of cofactors and vitamins”, and 8 pathways related to “Xenobiotic biodegradation and metabolism” (Welch’s *t* test, *p* < 0.01). On the other hand, the TTX group had higher abundances of 13 pathways related to “Carbohydrate metabolism”, 2 pathways related to “Glycan biosynthesis and metabolism”, 3 pathways related to “Lipid metabolism”, 6 pathways related to “Metabolism of terpenoids and polyketides”, and 1 pathway related to “Nucleotide metabolism” (Welch’s *t* test, *p* < 0.01) (Supplementary Fig. 8).

## Discussion

### Gut microbiota of juvenile *Takifugurubripes*

The gut bacterial community of juvenile pufferfish was studied, and the dominant phyla identified were Firmicutes, Campilobacterota, Spirochaetota, and Proteobacteria. The prevalence of Firmicutes and Campilobacterota was consistent with previous research, such as the reported relative abundance of Campilobacterota of 68.8% in pufferfish^33^. The predominant genera observed in the gut bacterial community were *Mycoplasma*, uncultured *Arcobacteraceae*, *Brevinema*, *Vibrio*, and *Rubritalea*, which comprised 95.7% of the community. These genera, along with uncultured *Pirellulaceae*, were identified as the core bacterial taxa, suggesting their vital role in maintaining the gut microbiome's overall stability and functionality. This finding aligned with earlier research on cultured juvenile tiger puffers^27^. Among the shared genera, *Mycoplasma*, uncultured *Arcobacteraceae*, and *Brevinema* exhibited persistently high relative abundances in the gut microbiota of juvenile pufferfish, regardless of whether the fish were fed a control diet or a TTX-containing diet. This observation suggested that these genera are robust and resilient, displaying a certain level of adaptability to environmental changes. Their consistent presence indicates a potentially intricate level of interdependence with the host, wherein they may play essential roles in gut microbiota stability and function. However, other studies have reported varying results regarding the most abundant genera in the pufferfish gut microbiota. Some studies have identified *Vibrio*, *Enterobacter*, and *Bacillus* as dominant genera^34^, while others have highlighted *Arcobacter*, *Photobacterium*, *Vagococcus*, *Weissella*, and *Vibrio*^33^. These discrepancies underscore the complexity of gut microbiota dynamics and highlight the need to understand the factors that determine the resilience and persistence of specific genera.

*Mycoplasma* dominance has been reported in various fish species, such as Atlantic salmon (*Salmo salar L.*)^79,80^, long-jawed mudsuckers (*Gillichthys mirabilis*)^81^, rainbow trout (*Oncorhynchus mykiss*)^82^, and TTX-bearing clams (*P. australis*)^83^. However, limited information is available on its functions and effects on the pufferfish gut microbiome. While *Mycoplasma* species are known for their minimal genomes and often rely on their host organisms for many nutrients, some strains may have limited biosynthetic capabilities. Additionally, certain Mycoplasma strains have been linked to tumors in fish^84^. A recent study showed that changes in gut microbes, including an increase in *Mycoplasma*, may be linked to increased activation of the host's energy and carbohydrate metabolic pathways^85^. Further study and verification are necessary to understand the functions and effects of *Mycoplasma* on *T. rubripes. Arcobacter* is a type of bacteria that is considered a potential cause of illness and a potential zoonotic agent^86^. However, it has been found to not cause any obvious disease symptoms in pufferfish, indicating that it may have other beneficial effects^33^. Under certain conditions, such as excess dietary astaxanthin and arachidonic acid levels, *Arcobacter* has been found to be dominant in the gut of pufferfish (salinity, 29–32‰)^27,28^. It has also been found to be dominant in the ulcer mucus surfaces of Atlantic salmon^80^, gut microbiota of hagfish^87^, and giant abalone (*Haliotis gigantea*)^88^. *Arcobacter* species have the metabolic capacity for sulfur oxidation coupled with N-oxide reduction while fixing carbon via the reverse TCA cycle^89–91^. Researchers have also speculated that this species may possess the ability to resist viruses, which could help to alleviate viral pressure^87^. *Brevinema,* a species of Spirochaetes, was commonly found in the gut microbiome of wild and farmed juvenile *Takifugu* species and could have played a beneficial role in the host^7,27,92,93^. It was also found in the digestive glands of TTX-bearing clams^83^. Spirochaetes are free-living bacteria that are abundant in marine environments^94^. Although some Spirochaetes contain pathogenic genera and species^95,96^, *Brevinema* can play a beneficial role in termites and other terrestrial herbivores through acetogenesis and dinitrogen fixation^97–99^. Spirochaetes have also been suggested to play important ecological roles, such as nitrogen fixation, in herbivorous fishes^100–102^. *Vibrio* was found to be the most dominant genus in the gut microbiome of both the 30_Control and 1.7_Control groups, with percentages of 9.8% and 7.7%, respectively. This finding was consistent with previous studies on marine species such as pufferfish^27,29,33^, Japanese coastal fishes^103^, and ayu (*Plecoglossus altivelis*)^104^. Although some species of *Vibrio* can be harmful, many are harmless and naturally occur in aquatic environments with salinity levels of 29–32%. *Vibrio* spp. are also capable of producing hydrolytic enzymes such as amylase, lipase, cellulase, and chitinase^105^. *Rubritalea* is a marine bacterium found in various environments, such as the Mediterranean sponge *Axinella polypoides*^106^, juvenile *Lates calcarifer*^107^, and shrimp infected with White Feces Disease^108^. Certain strains of *Rubritalea* produce carotenoid compounds, resulting in the pink coloration of the cellular biomass^109^. Additionally, some strains can produce squalene compounds^110^. *Pirellulaceae* is a family of aquatic bacteria found in both marine and freshwater environments. *Pirellulaceae* was previously found in the gut microbiome of Nile tilapia^111^ and the intertidal barnacle *Semibalanus balanoides*^112^ and has been identified in association with soils^113^. *Pirellula*-like planctomycetes are ubiquitous aquatic bacteria that are often detected in anoxic or microoxic habitats^114^. Although the precise role of the family *Pirellulaceae* is not fully understood, a previous study suggested that heterotrophic Planctomycetota play a significant role in the fermentation of carbohydrates^115^. The identification of this taxon as a core component of the microbiota within the intestinal tract of *T. rubripes* implies that it may play a role in the breakdown and digestion of consumed carbohydrates.

### Influence of TTX on the gut microbiota of juvenile *Takifugurubripes*

Our study aimed to explore how TTX administration affects the gut microbiota of *T. rubripes* reared under varying salinity conditions. We did not specifically address the potential influence of feed microbes, both with and without TTX treatment, on the gut microbiota in this study. However, in a separate experiment focusing on feed microbiota analysis, we found no significant differences between feeds with and without TTX, suggesting that feed microbiota variability may not be a major factor contributing to differences in the gut microbiota (data not shown). Additionally, we observed variations in body weight among fish reared at different salinity levels. It's important to note that changes in salinity, body weight, and/or age of the fish could potentially impact the gut microbiota composition. However, further experiments are required to specifically evaluate the effects of different salinity levels on the gut microbiota of the same fish batch. Therefore, to compare microbiota differences, we focused solely on comparing control fish and TTX-fed fish reared at the same salinity level, as no significant difference in body weight was observed between these groups.

We found no significant differences in high-abundance genera between TTX-fed fish and control fish at either the 30.0 ppt or 8.5 ppt salinity level (Fig. 2B,C). Additionally, the alpha and beta diversity of the gut microbiota did not differ significantly between puffers fed TTX-containing diets and those fed control diets at the same salinity level (Fig. 3). Certain high-abundance genera, such as *Mycoplasma*, uncultured *Arcobacteriaceae*, *Brevinema*, *Vibrio*, and *Rubritalea*, may be insensitive to TTX and thus remain relatively stable. Previous research has shown that TTX primarily targets mammalian voltage-gated sodium (Na_v_) channels but fails to affect some bacterial sodium channels^41,42^. Bacterial sodium channels, such as *Bacillus halodurans* NaChBac and *Arcobacter butzleri* Na_v_Ab, are insensitive to TTX, even at micromolar toxin concentrations^41^. The resistance of Na_v_Ab to TTX is attributed to the inability of the TTX guanidine to form strong interactions with the channel in the presence of two Na + ions^41,42^. This finding may provide a possible explanation for why bacteria such as *Mycoplasma*, uncultured *Arcobacteraceae*, and *Brevinema* could persist in the gut microbiota of pufferfish, potentially exhibiting TTX resistance.

However, we observed statistically significant differences in the relative abundances of certain ASVs between TTX-fed and control fish reared at various salinity levels (Figs. 4 and 5), suggesting that TTX ingestion has a noticeable impact on the composition of the gut microbiota. These findings indicate that TTX ingestion can lead to alterations in the relative abundances of specific taxa within the gut microbiota community. This change may result in the inhibition of susceptible taxa and the proliferation of more resistant taxa. While TTX is primarily known for its neurotoxic effects on nerve cells^1,3,4^, accumulating evidence suggests that TTX can also affect microbial cells^39,40^. Therefore, direct exposure to TTX can lead to cell death or disruption of microbial metabolism, directly influencing microbial taxa within the gut microbiota. Alternatively, TTX ingestion can induce physiological changes in the host organism^45^, which in turn can influence the gut environment and microbial taxa composition. For instance, TTX may alter gut motility, the secretion of digestive enzymes, or immune responses in the host, creating conditions that favor the growth of certain microbial taxa over others. We observed a significant increase in the abundance of uncultured *Arcobacteraceae* in fish that were fed TTX, particularly at 1.7 ppt salinity (Fig. 2A). Additionally, plasma cortisol levels were significantly higher in fish reared at 1.7 ppt salinity (Supplementary Fig. 2C), indicating a stress response in the host. Stress-induced changes in the gut environment can influence the diversity and abundance of microbial communities, potentially shaping the gut microbiota composition in response to stress. *T. rubripes* might selectively promote the growth of some *Arcobacter* spp. to cope with such drastic changes in the microbiota. This adaptation could supply essential compounds to support the growth of juveniles at stressful salinity levels. TTX administration might enhance the proliferation of *Arcobacter* spp. and other residential bacteria by inhibiting the growth of other bacteria. Previous research has suggested that the abundance of *Arcobacter* may be beneficial for the host rather than pathogenic, due to its metabolic capabilities for sulfur oxidation and carbon fixation, as well as its ability to resist viruses in hagfish^87,89–91^. In our study, fish fed a TTX-containing diet displayed the highest body weight at 1.7 ppt salinity. A previous study revealed that TTX-fed fish exhibited the greatest growth at 8.5 ppt salinty^45^. Another study indicated that juvenile *T. rubripes* fed a TTX-containing diet exhibited greater growth than those fed a control diet, suggesting that TTX has a positive effect on the growth of juvenile tiger puffer^36^. Researchers have also shown that TTX administration can improve the survival and growth performance of fish by reducing stress and activating the hypothalamo–pituitary–interrenal (HPI) axis^36^. As the gut is a primary site for microbial colonization, changes in the physiological environment of the intestine can influence the composition and dynamics of the gut microbiome.

### The influence of TTX on the functional profiles of the gut microbiome of juvenile *T. rubripes*

The use of Tax4fun to predict the functional profiles of the juvenile *T. rubripes* gut microbiome provided insights into the possible effects of a shift in ASVs caused by TTX administration on metabolic capabilities. Our results from the heatmap, LEFSe, and Venn diagram showed variations in ASVs, which were reflected in the differences in functional profiles of the gut microbiota between the control and TTX-fed fish. The investigation revealed distinct microbial responses to TTX ingestion, with differences observed in various functional categories. Notably, TTX ingestion had an impact on the “Carbohydrate metabolism,” “Glycan biosynthesis and metabolism,” “Lipid metabolism,” “Metabolism of terpenoids and polyketides,” and “Nucleotide metabolism” categories, exhibiting high abundances in TTX-fed fish. These findings suggest that the diverse metabolic activities of the microbiota were possibly caused by TTX ingestion. TTX, which is known to be absorbed from the intestine through saturable mechanisms^116^, could influence the microbiota residing in the intestine and affect the intricate balance of microbial communities. Moreover, these changes could transmit signals to the brain, possibly acting as a stress-relieving substance. This connection between TTX, the gut microbiota, and the stress response adds a layer of complexity to our understanding of the physiological adaptations of *T. rubripes* to environmental challenges. Further exploration of these interactions could reveal novel insights into the intricate dynamics of fish–microbiota interactions and stress regulation in this unique species.

## Conclusions

Our study aimed to investigate whether administering TTX to fish could alter their gut microbiota and influence their overall health. We discovered that the dominant phyla of the juvenile *T. rubripes* gut microbiome were Firmicutes, Campilobacterota, Spirochaetota, and Proteobacteria, with core bacterial genera such as *Mycoplasma*, uncultured *Arcobacteraceae*, *Brevinema*, *Vibrio*, *Rubritalea*, and uncultured *Pirellulaceae*. After TTX was administered, the relative abundances of these core taxa remained stable under normal salinity conditions (8.5 ppt and 30.0 ppt) but changed at a stressful salinity level (1.7 ppt). We observed an increase in the relative abundance of uncultured *Arcobacteraceae* at a stressful salinity level after TTX administration. This genus is believed to be beneficial for the host, suggesting its potential as a probiotic candidate for *T. rubripes*. Additionally, TTX administration led to changes in the bacterial community, particularly among low-abundance taxa, affecting the overall functions of the gut microbiota under all salinity conditions. These changes have significant implications for the health and survival of juvenile *T. rubripes.* In the future, we aim to understand the reason behind the assembly of the core microbial taxa identified in this study and to examine whether rearing water and feed are related to the dynamics of the gut microbiome. Additionally, we aim to delve deeper into the functional roles of the *T. rubripes* gut microbiota, particularly focusing on the uncultured *Arcobacteraceae.* We will examine how the *T. rubripes* gut microbiota responds to TTX through gene expression studies and assess the direct effects of TTX on the growth performance of specific species isolated from the *T. rubripes* gut microbiota. These studies are essential for a thorough understanding of these complex dynamics.

### Supplementary Information

Supplementary Information.

## Data Availability

The datasets presented in this study can be found in online repositories. The names of the repositories and accession number(s) can be found at https://www.ddbj.nig.ac.jp/, DRA016756.
