# Supplementary material for: The impact of tetrodotoxin (TTX) on the gut microbiome in juvenile tiger pufferfish, Takifugu rubripes
Source: Sci Rep. 2024 Jul 31;14:16684. doi: 10.1038/s41598-024-66112-y (PMC11291987; doi:10.1038/s41598-024-66112-y)
Supplement: Supplementary file 1 — Supplementary Information. [file 41598_2024_66112_MOESM1_ESM.pdf]

Supplementary Material

The Impact of Tetrodotoxin (TTX) on the Gut Microbiome in Juvenile Tiger Pufferfish, *Takifugu rubripes*

Mai A. Wassel[1,2]*, Yoko Makabe-Kobayashi[1], Md Mehedi Iqbal[1], Tomohiro Takatani[3], Yoshitaka Sakakura[3], Koji Hamasaki[1,4,5]*

1 Supplementary Figures

Supplementary Figure 1. Diagrammatic summary of the experimental procedure used to control salinity and diet of *T. rubripes* juveniles.

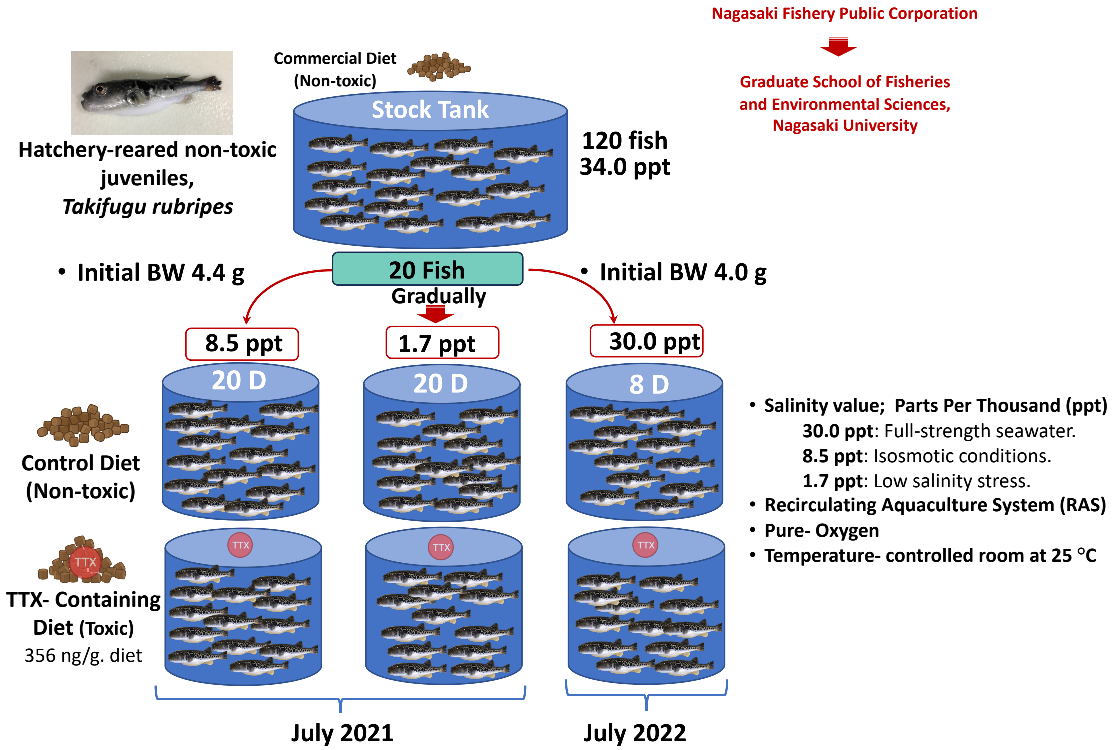

Supplementary Figure 2. Physiological parameters of juvenile *T. rubripes* reared at three different salinities for 20 days (1.7 ppt & 8.5 ppt), and 8 days (30.0 ppt), fed diets with and without TTX: **(A)** body weight (g); **(B)** plasma osmolality; **(C)** plasma cortisol levels; and **(D)** plasma lysozyme activity.

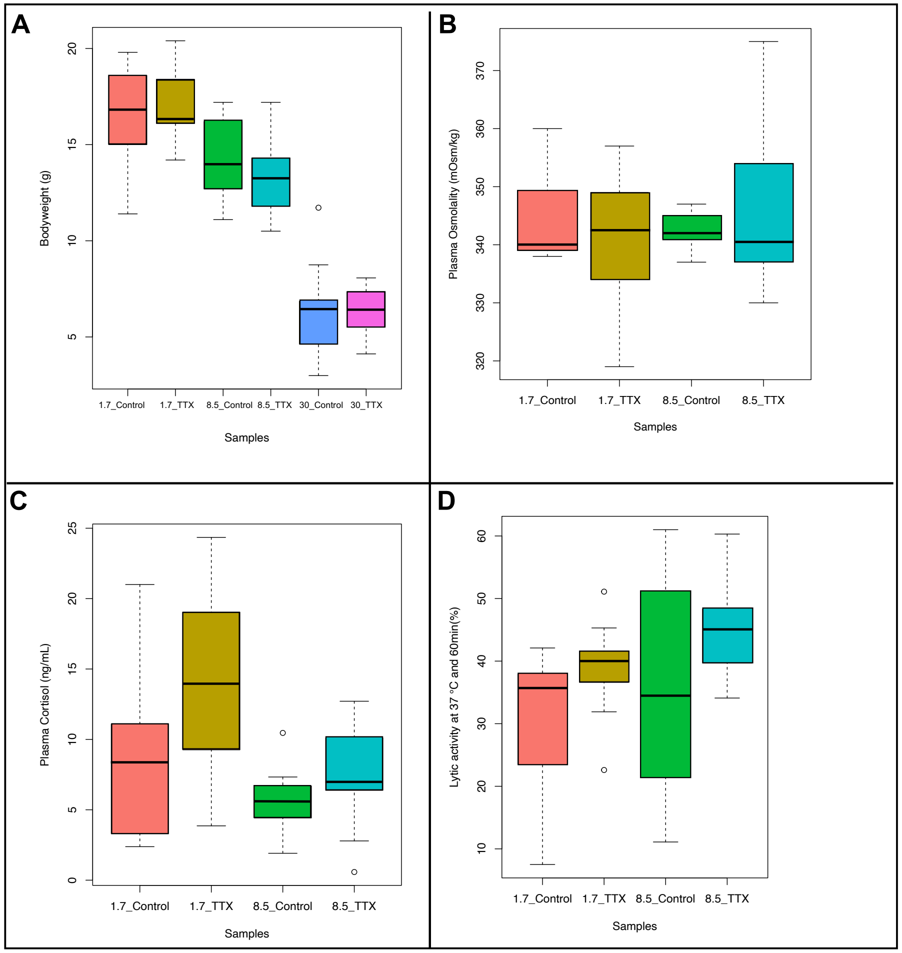

Supplementary Figure 3. Heatmap displaying the top 50 genera of gut bacterial communities in *T. rubripes* juveniles, comparing control fish to TTX-fed fish at the genus level.

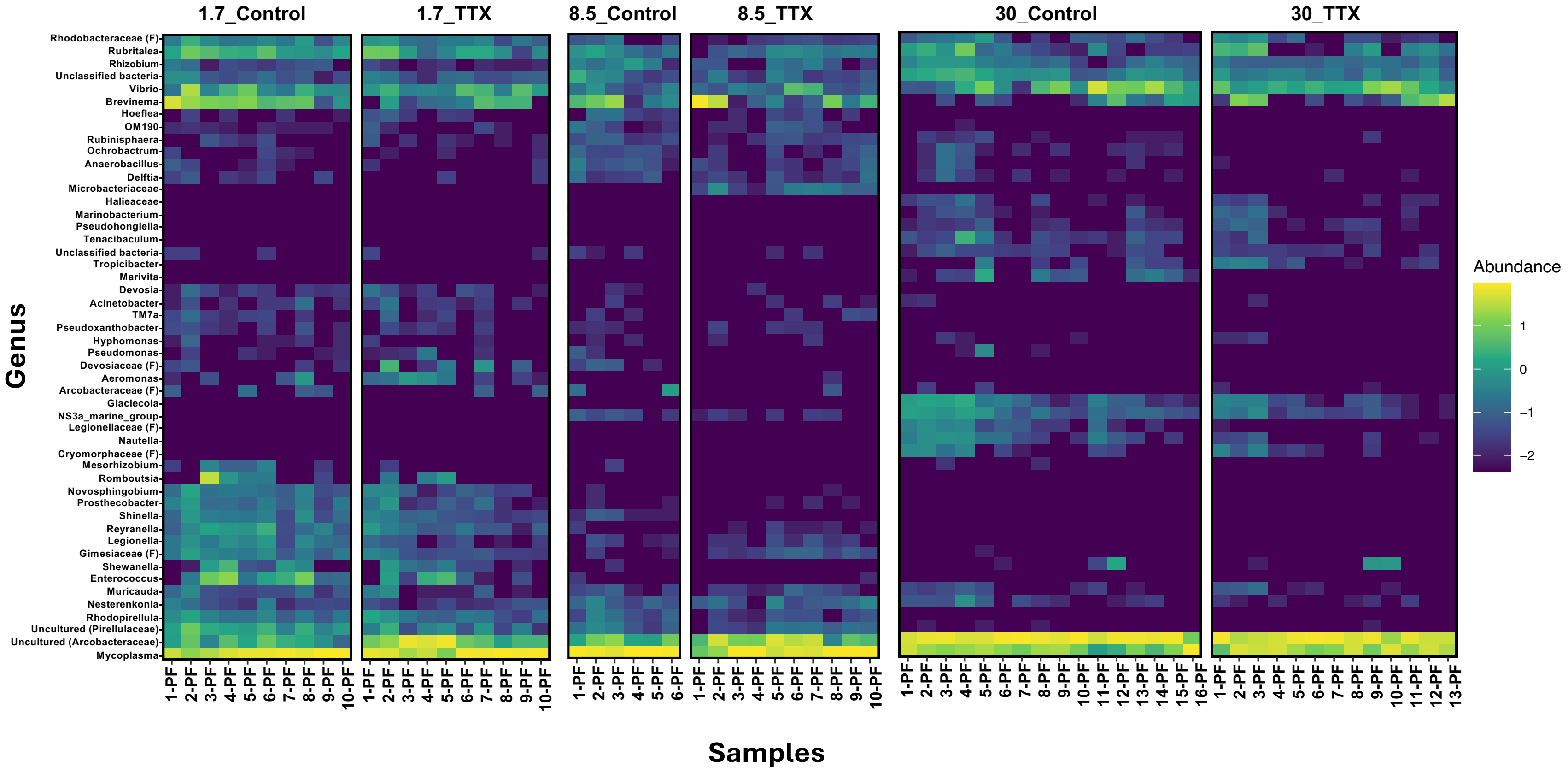

Supplementary Figure 4. Venn diagram illustrating the shared and unique bacteria at the ASV level among control fish and TTX-fed fish reared at three different salinity levels: **(A)** 1.7_Control & 1.7_TTX groups; **(B)** 8.5_Control & 8.5_TTX groups; and **(C)** 30_Control & 30_TTX groups.

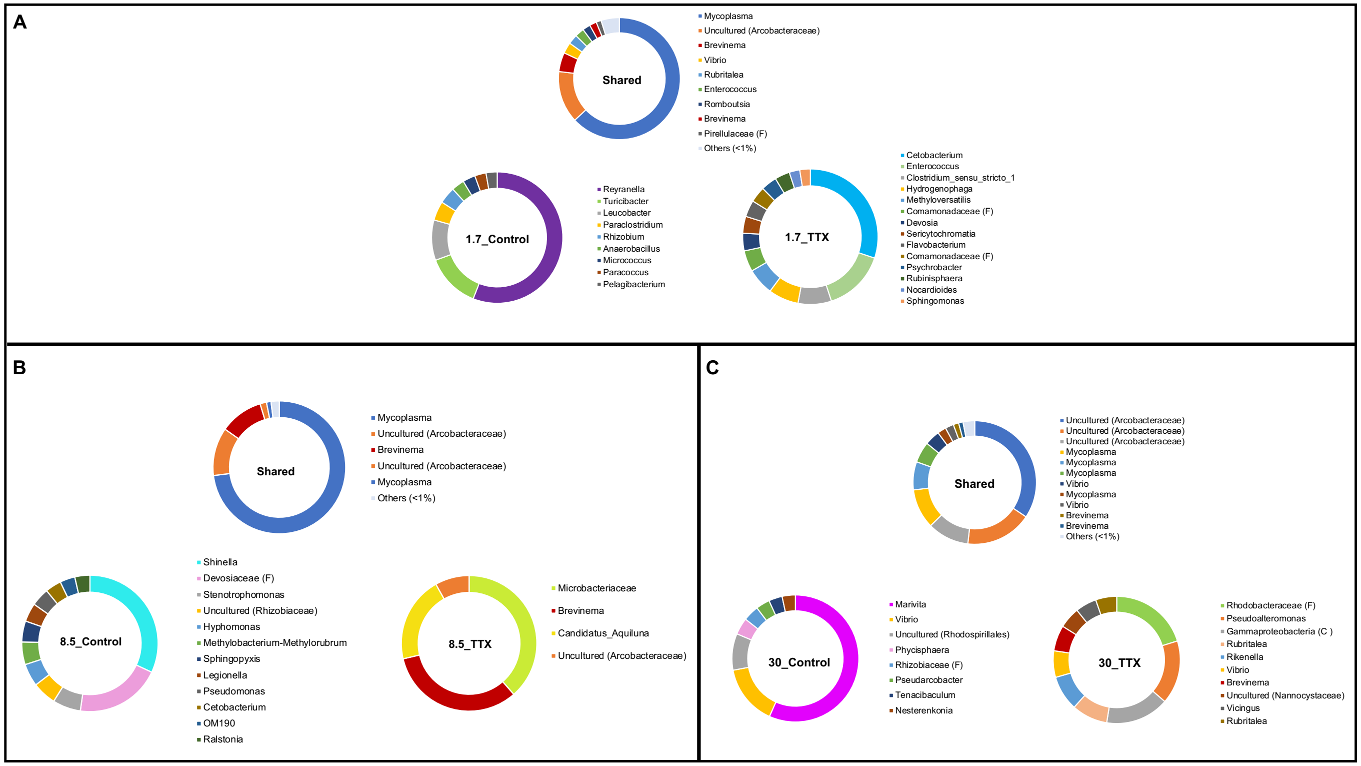

Supplementary Figure 5. The interactive heatmap depicts the relative abundance of KEGG metabolism (level 2) within the gut bacterial community in *T. rubripes* at three different salinity levels fed diets with and without TTX: **(A)** 1.7_Control & 1.7_TTX groups; **(B)** 8.5_Control & 8.5_TTX groups; and **(C)** 30_Control & 30_TTX groups. https://www.kegg.jp/kegg/kegg1.html

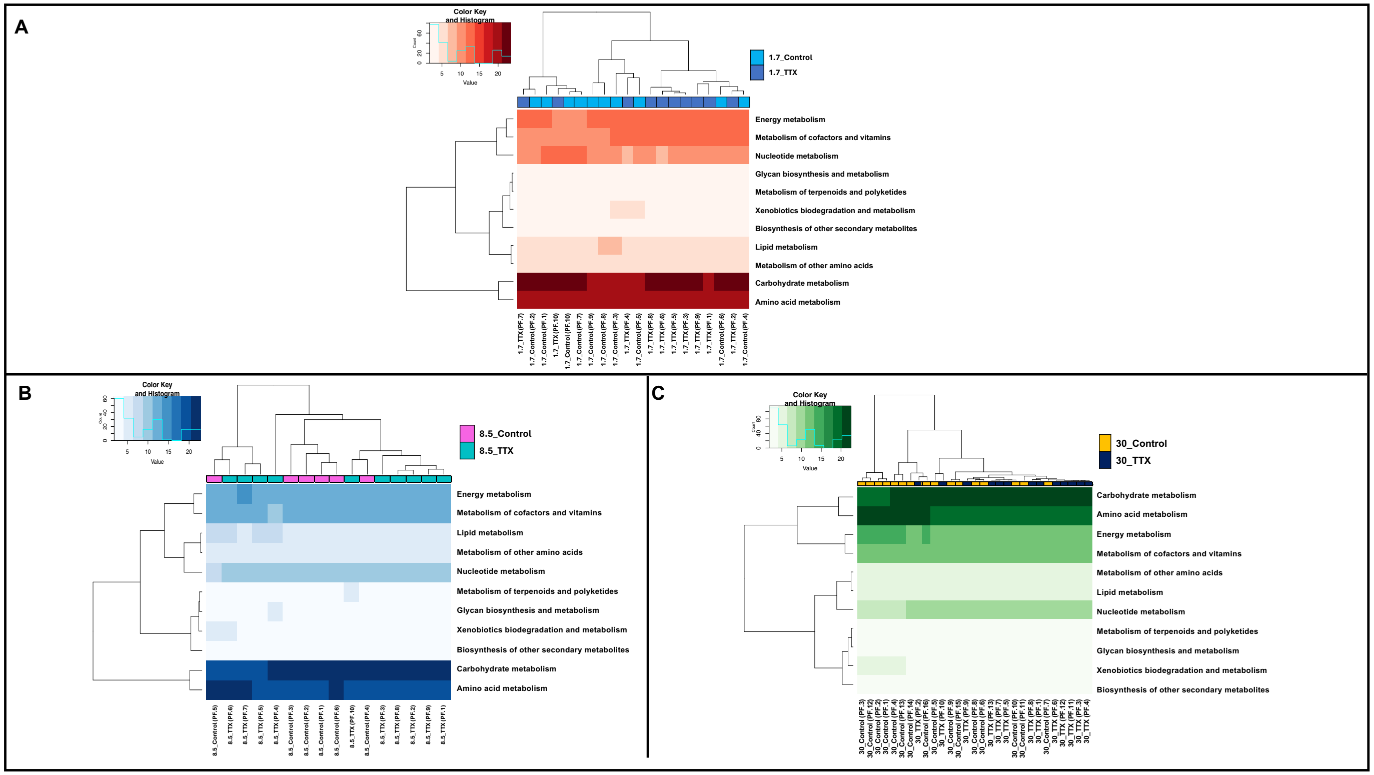

Supplementary Figure 6. Relative abundance of the KEGG categories (level 3) of gut bacterial community in control fish and TTX-fed fish at salinity level of 1.7 (Welch's t test, $p < 0.05$). https://www.kegg.jp/kegg/kegg1.html

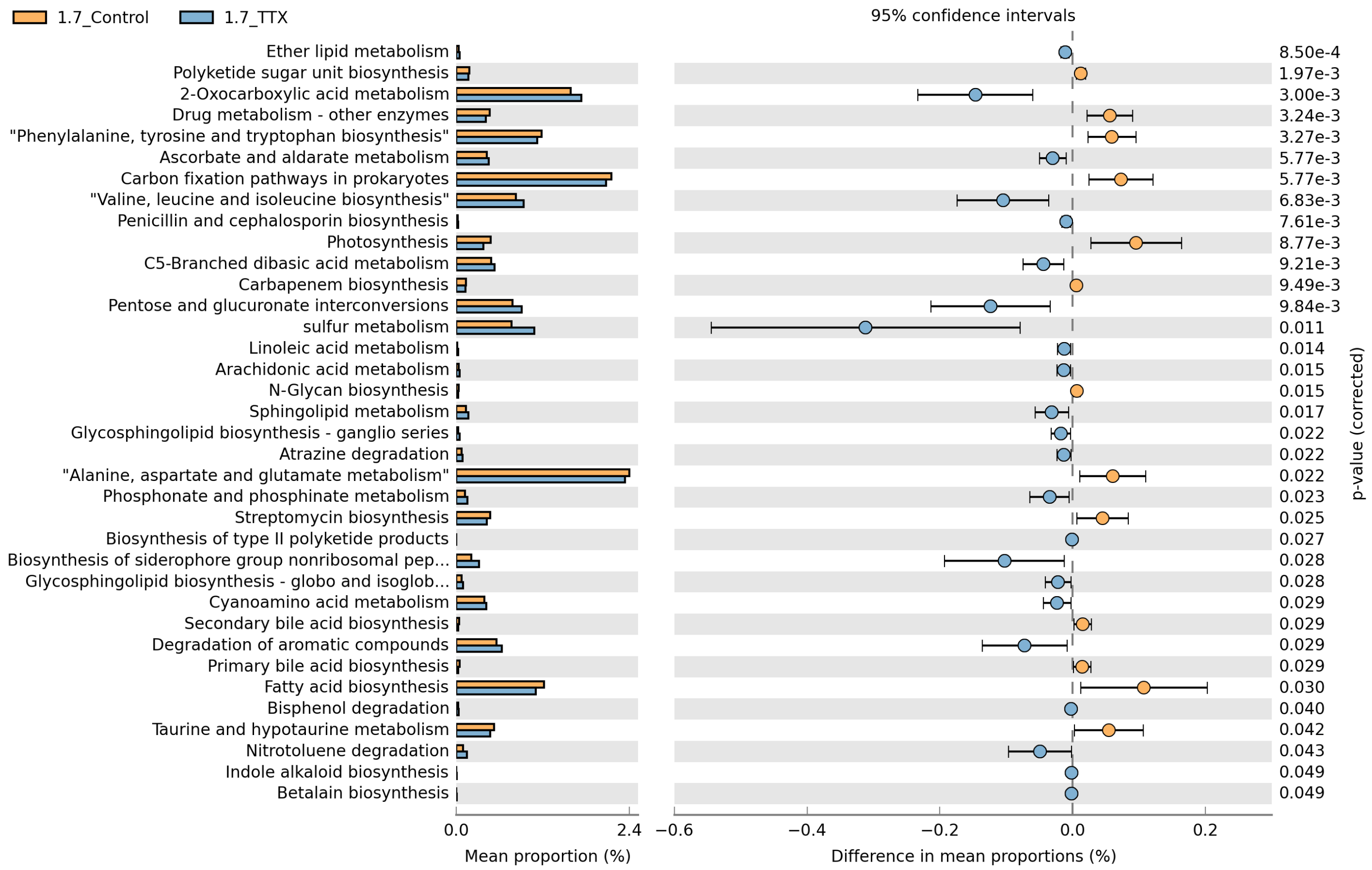

Supplementary Figure 7. Relative abundance of the KEGG categories (level 3) of gut bacterial community in control fish and TTX-fed fish at salinity level of 8.5 (Welch's t test, $p < 0.05$). https://www.kegg.jp/kegg/kegg1.html

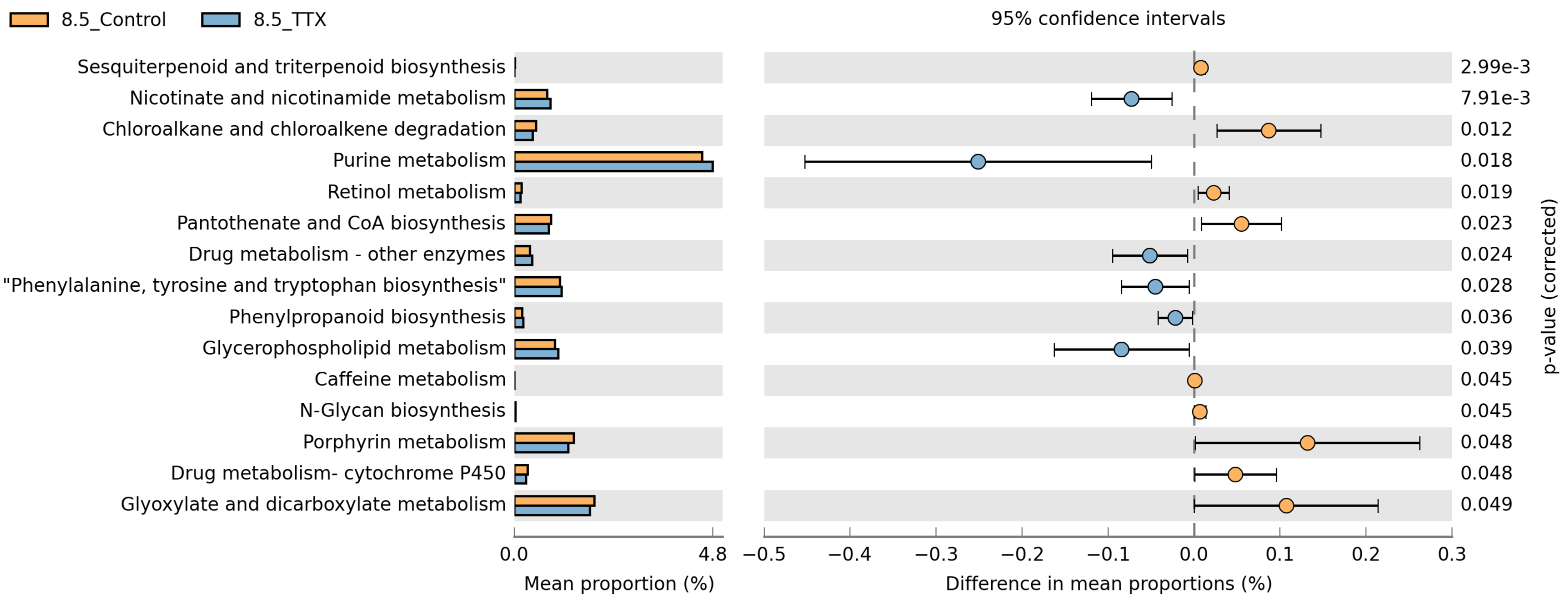

Supplementary Figure 8. Relative abundance of the KEGG categories (level 3) of gut bacterial community in control fish and TTX-fed fish at salinity level of 30.0 (Welch's t test, $p < 0.05$). https://www.kegg.jp/kegg/kegg1.html

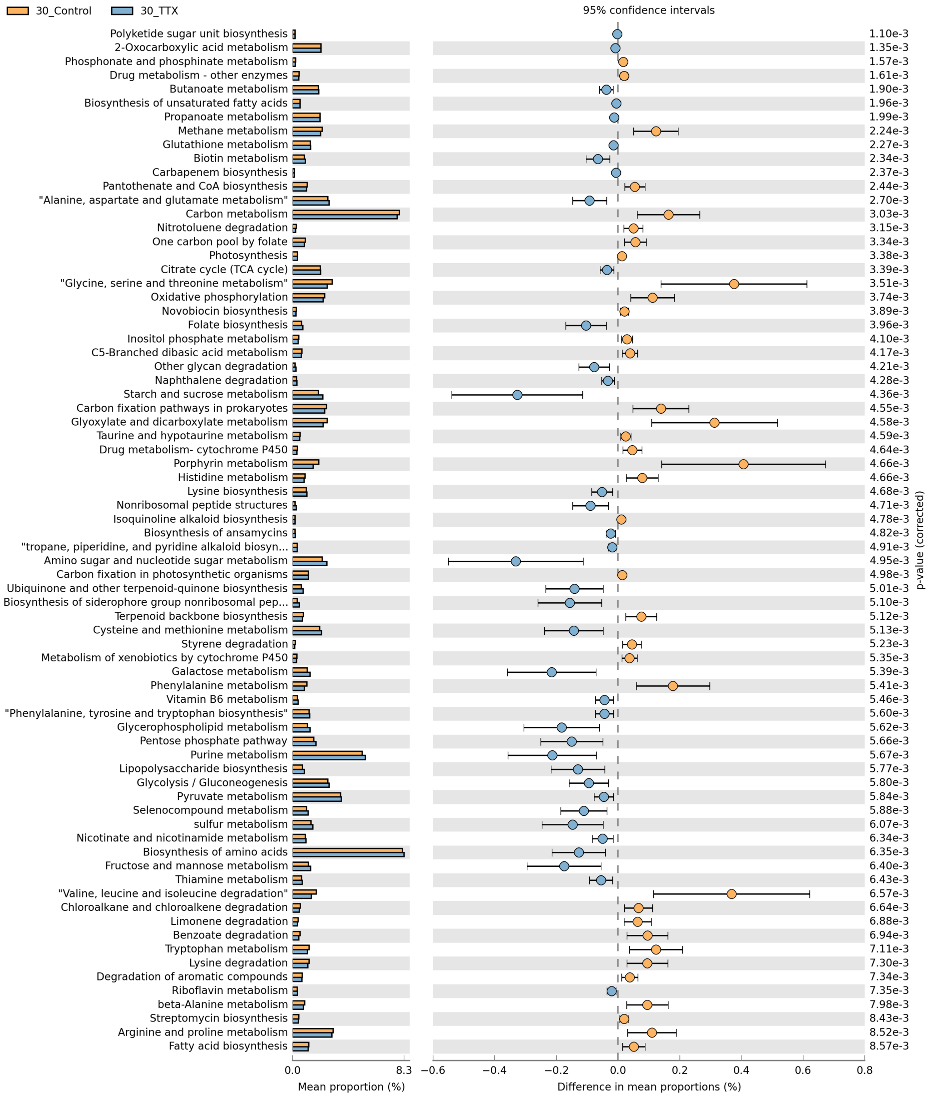

Supplementary Figure 9. Rarefaction curves were generated based on observed amplicon sequence variants (ASVs) and Shannon diversity index measure for each sample of *T. rubripes* juveniles in two groups—control fish and TTX-fed fish—at three different salinity levels: 1.7 ppt (low-salinity stress), 8.5 ppt (isosmotic), and 30.0 ppt (full-strength seawater). Samples are color-coded by each sample in each treatment.

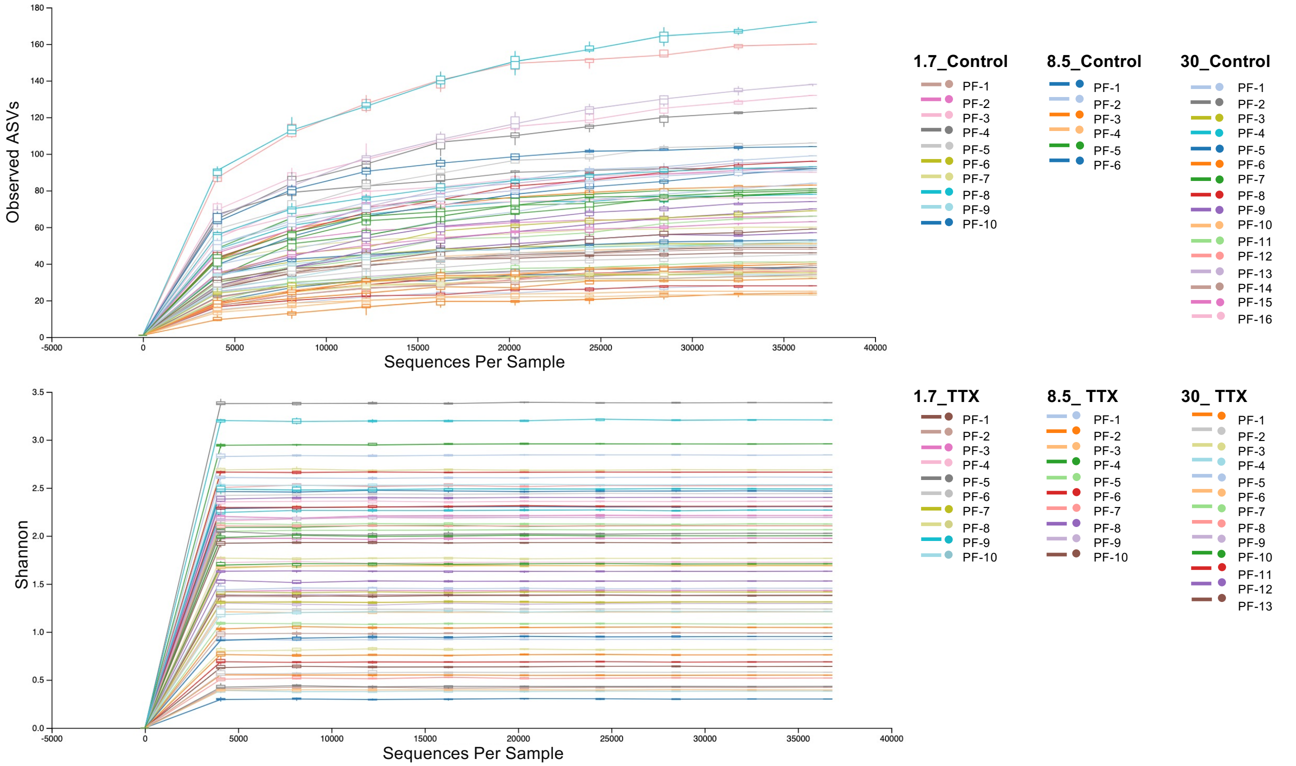

2 Supplementary Tables

Supplementary Table 1. Growth of juvenile *T. rubripes* reared at three different salinities; 1.7 ppt & 8.5 ppt for 20 days and 30.0 ppt for 8 days, fed diets with and without TTX (mean ± SD)

Salinity	Diet	n	SL (mm)	BW (g)
1.7 ppt	Control	10	79.2 ± 3.9	16.1 ± 2.5
8.5 ppt	Control	6	75.4 ± 4.1	14.1 ± 2.1
30.0 ppt	Control	16	59.2 ± 6.4	6.2 ± 2.2
1.7 ppt	TTX	10	82.0 ± 3.8	17.0 ± 1.9
8.5 ppt	TTX	10	74.9 ± 3.6	13.3 ± 2.0
30.0 ppt	TTX	13	59.2 ± 3.8	6.3 ± 1.2

Supplementary Table 2. TTX accumulation in juvenile *T. rubripes* reared at two different salinities; 1.7 ppt & 8.5 ppt for 20 days, fed diets with and without TTX (mean ± SD).

Salinity (ppt)	Food	n	Tetrodotoxin levels			
			Liver (MU/g)	Muscle (MU/g)	Skin (MU/g)	Tissue (MU/tissue)
1.7	Control	10	N.D	N.D	N.D	N.D
8.5	Control	6	N.D	N.D	N.D	N.D
1.7	TTX	9	N.D	N.D	0.2 ± 0.1	0.6 ± 0.3
8.5	TTX	10	N.D	N.D	N.D	N.D

N.D: not detected
